# Interferon activation in bone marrow long-lived plasma cells in systemic lupus erythematosus

**DOI:** 10.3389/fimmu.2024.1499551

**Published:** 2025-01-10

**Authors:** Diana F. Alzamareh, Nida Meednu, Neha Nandedkar-Kulkarni, Daria Krenitsky, Jennifer Barnard, Ken Yasaka, Wesley Durrett, Juilee Thakar, Javier Rangel-Moreno, Jennifer H. Anolik, Jennifer L. Barnas

**Affiliations:** ^1^ Division of Allergy, Immunology and Rheumatology, University of Rochester Medical Center, Rochester, NY, United States; ^2^ Department of Biostatistics and Computational Biology, University of Rochester Medical Center, Rochester, NY, United States; ^3^ Department of Microbiology and Immunology, University of Rochester Medical Center, Rochester, NY, United States

**Keywords:** plasma cell (PC), lupus (SLE), transitional B cell, bone marrow, interferon, B cell, anifrolumab, verdinexor

## Abstract

While durable antibody responses from long-lived plasma cell (LLPC) populations are important for protection against pathogens, LLPC may be harmful if they produce antibodies against self-proteins or self-nuclear antigens as occurs in autoimmune diseases such as systemic lupus erythematosus (SLE). Thus, the elimination of autoreactive LLPC may improve the treatment of antibody-driven autoimmune diseases. However, LLPC remain a challenging therapeutic target. Here, we compare the matched bone marrow (BM) and peripheral blood (PBL) plasma cell (PC) compartments of SLE and healthy donors (HD). We show a similar distribution of CD138- and CD138+ PC, including putative LLPC (CD19- CD138+ CD38+), between SLE and HD BM. For both SLE and HD, CD138+ PC are at a higher frequency in BM than PBL. Expression of Ki-67 associates with the PBL compartment where it is found on all PC subsets regardless of CD19 or CD138 expression. Transcriptomic analysis identifies an interferon (IFN) gene signature in transitional B cells in the SLE BM, but surprisingly also in the BM PC derived from SLE. BM PC and B cells phosphorylate STAT1 in response to type I IFN stimulation *in vitro*, but with decreased fold change compared to those from the PBL. While BM PC bind type I IFN receptor-blocking antibody anifrolumab, it is to a lesser degree than circulating B cells. Anti-nuclear autoantibodies (ANA) are found in the BM supernatant and PBL serum of SLE patients. Both SLE and HD BM-derived PC have increased survival compared to their PBL counterparts when treated with verdinexor. In summary, these findings show evidence of IFN activation in BM PC from SLE.

## Highlights

- ANA are found in lupus bone marrow.
- SLE BM PC express a type I IFN gene signature.
- BM PC subset distribution was similar in SLE and HD.
- BM PC are more resistant to cell death compared to PBL PC.
- Signaling is activated in PC and B cells in response to IFN-α.

## Introduction

Systemic lupus erythematosus (SLE) is an autoimmune disease defined by the presence of anti-nuclear autoantibodies (ANA), which contribute to multi-system end organ damage. Plasma cells (PC)–the terminal differentiation state of the B lymphocyte lineage–are responsible for the sustained production of immunoglobulin. Increases in circulating plasmablasts (PB) are associated with SLE disease flares (1, 2). Evidence of PC longevity can be seen in human transplant patients, where donors’ long-lived plasma cells (LLPC) are found decades later in pancreas transplant recipients receiving adjacent duodenum (3). Subsets of PC migrate to the bone marrow (BM) where they find a survival niche. Their extraordinary longevity provides decades of serological memory and thus confers protection against pathogens via production of antibodies. In autoimmunity, autoantibodies made by LLPC might be responsible for the persistence of disease and their elimination may represent a potential cure. If unique features could be identified in the autoimmune BM PC, those features might serve as therapeutic targets.

During a normal immune response in mice, large foci of B cells form in the spleen that then reduce in size until nearly absent two weeks later (4). From these foci, PC are generated which peak in number around 1 week after antigen exposure (4). The majority of PC will die, but a subset changes their chemokine responsiveness and traffic to the BM (5, 6). Within the BM, accessory cells support PC survival [reviewed in (7, 8)] including CXCL12-producing stromal cells, eosinophils (9), basophils (10), T regulatory cells (11), monocytes (12), and megakaryocytes (13). In culture, reproducing BM niche factors–hypoxia, mesenchymal stem cell-secreted factors, and APRIL– extend the life span of PCs from hours to up to 50 days (7). Thus, the BM microenvironment acts as a reservoir for LLPC and thus long-term antibody production (14).

In autoimmunity, the LLPC are a source of autoantibodies. LLPC from NZB/W lupus-prone mice produce anti-dsDNA autoantibodies which lead to lupus nephritis in their original NZB/W donors and in normal mice when NZB/W LLPC are adoptively transferred (15, 16). Murine autoreactive LLPC are found in the spleen and BM (15, 16), as well as in the inflamed kidney in lupus nephritis (17). LLPC in humans are contained within the CD19- CD38+ CD138+ compartment (18) and have been proposed as a therapeutic target for human SLE.

Here, we characterize peripheral blood (PBL) and BM PCs from both SLE and healthy donors (HD) to understand factors that contribute to PC longevity and autoreactivity in SLE and to characterize how PC derived from the PBL differ from those of the BM compartment. We find increased numbers of LLPC in the BM compared to PBL, but PBL PC were more proliferative regardless of phenotype. Both SLE BM LLPC and short-lived PC (SLPC) upregulate type I interferon (IFN) pathways when compared to HD and type I IFN was detected in BM supernatant. These cells respond to IFN-α as shown at the single cell level by phosphorylation of STAT1. We detect ANA in human SLE BM supernatant which correlates detection of ANA in PBL serum. We also find that BM PCs are a challenging target even *ex vivo* as the BM derived PCs show enhanced survival over PBL PCs with selective inhibitor of nuclear transport [SINE, reviewed in (19)] verdinexor treatment, as well as decreased binding of anifrolumab (a type I IFN receptor blocking antibody approved for treatment of SLE) compared to PBL PCs.

## Materials and methods

### Patients and samples

**Figure 1:**
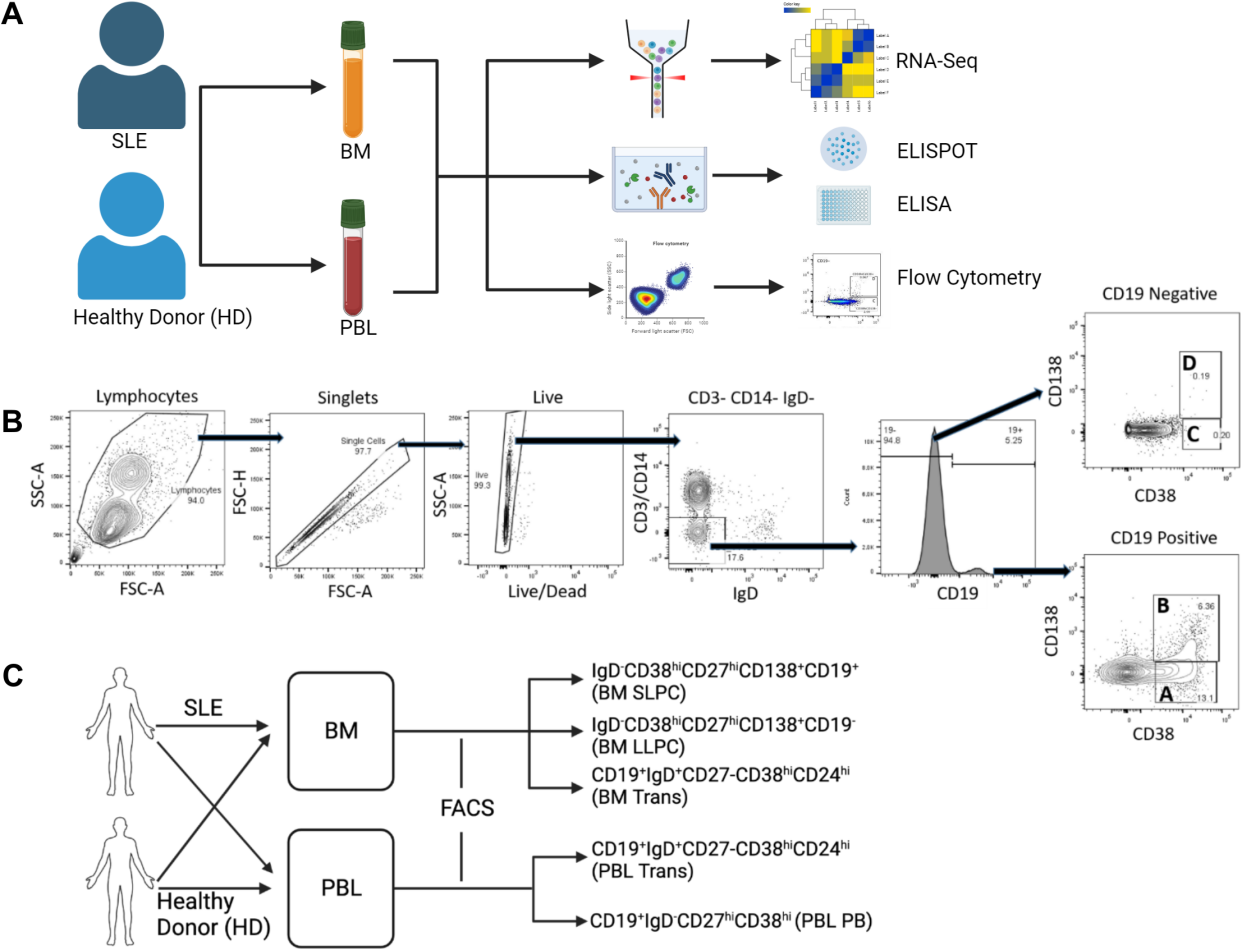
Experimental Overview. **(A)** Paired PBL and BM from HD or SLE donors were subjected to RNA-sequencing transcriptomic analysis, IgG ELISPOT, IFN ELISA and/or immunophenotyping by flow cytometry. **(B)** Representative flow cytometry PC population gating strategy. A sample derived from PBMC is shown. (B & C). PC fractions A, B, C, D are phenotyped in **Figures 2**, **3**. **(C)** Sorting Strategy. Paired PBMC and BMMC were sorted into putative BM LLPC, BM SLPC, BM transitional B, PBL PB, and PBL transitional B cells by fluorescence activated cell sorting. Bulk transcriptomic analysis performed on these samples is shown in **Figures 4**–**7**. Figure made with BioRender.com.

Samples were collected from SLE and HD via venipuncture at the University of Rochester Medical Center or affiliated outpatient clinics after informed consent in accordance with approved Institutional Review Board protocol. All patients fulfilled 1997 or 2019 ACR SLE classification criteria (20, 21). Disease activity was assessed by the Systemic Lupus Erythematosus Disease Activity Index (SLEDAI)-2K (22). All patients were on pharmacologic therapy for lupus. All patients had a positive ANA (indirect immunofluorescence with Hep-2 cells) of at least 1:80 at least once. Anti-dsDNA titers were determined by the clinical laboratory. Anticoagulated venous PBL or BM from SLE patients and HD was subjected to density gradient centrifugation to isolate PBL or BM mononuclear cells (MC). RNA-seq, *ex vivo* phenotyping, phosphorylation flow cytometry and cultured experiments utilized fresh cells without freezing. Samples for anifrolumab phenotyping were stored in liquid nitrogen and rapidly thawed in batches for analysis. Serum and BM supernatant were stored at -80°C. Most subjects were used for multiple assay types (**Figure 1A**).

### Flow cytometry phenotyping and sorting

PBL and BM were obtained from 7 HD and 9 SLE patients for immunophenotyping. Cell viability was determined by incubation of cells with LIVE/DEAD™ Fixable Aqua Dead Cell Stain Kit (Invitrogen, Carlsbad, CA, USA, catalog L34966) following the manufacturer’s protocol. Samples were incubated with antibodies for 30 minutes at 4°C. Intracellular staining was performed using eBioscience FoxP3/Transcription Factor Staining Buffer Set (Invitrogen, catalog 00-5523-00) following the manufacturer’s recommendations. Cells were fixed for 20 minutes in 1% methanol-free formaldehyde then diluted in 1% BSA in PBS in a 2:1 ratio. Cells were run on an LSR II Fortessa flow cytometer (BD). Data analysis was performed using FlowJo software (USA). The following monoclonal anti-human antibodies were used for B cell phenotyping: CD28-BB515 (clone CD28.2, BD 564492), CD20-biotin (clone 2H7, Invitrogen, 13-0209), CD3-Alexa700 (clone SP34.2, BD 557917), CD24-BUV395 (clone ML5, BD 563818), IgG-PE-Cy7 (clone G18-145, BD 561298), IgM-BV605 (clone G20-127, BD 532977), IgA-APC (clone IS11-8E10, Miltenyi 130-113-472), Ki-67-PD (clone B56, BD 556027), CD27- BUV737 (clone L128, BD 612829), CD138-Dazzle (clone M115, BL 356530), CD38-BB700 (clone HIT2, BD 566445), CD14-Alexa700 (clone M5E2, BD 557923), IgD-BV421 (clone IA6-2, BL 348226), streptavidin-PE-Cy5 (Invitrogen 15-4317). PCs were defined as CD3- CD14- IgD- CD38+ and divided into subsets based on CD19 and CD138 expression. Putative LLPC of the bone marrow express CD138 but no CD19, whereas SLPC expressed CD19 and CD138 (**Figure 1B**).

### Bulk RNA-seq

HD and SLE PBL and BM MC were FACS sorted as PBL or BM transitional B cells, CD19+ BM PC, CD19- BM PC, and PBL PB (**Figure 1C**) into Qiagen RLT lysis buffer. Total RNA was isolated using the RNeasy Plus Micro Kit (Qiagen, Valencia, CA) per the manufacturer’s recommendations. RNA concentration was determined with the NanoDrop 1000 spectrophotometer (NanoDrop, Wilmington, DE), and RNA quality assessed with the Agilent Bioanalyzer 2100 (Agilent, Santa Clara, CA). The Total RNA (1 ng) was pre-amplified with the SMARTer Ultra Low Input kit v4 (Clontech, Mountain View, CA) per manufacturer’s recommendations. The quantity and quality of the subsequent cDNA were determined using the Qubit Fluorimeter (Life Technnologies, Carlsbad, CA) and the Agilent Bioanalyzer 2100 (Agilent, Santa Clara, CA). High-quality RNA was obtained from 6 SLE and 5 HD. There were too few PBL transitional B cells in any of the HD and 1 SLE donor to obtain sufficient RNA for sequencing. Quality RNA was only available for BM transitional B cells for 5 out of the 6 SLE patients. PBL PB RNA was isolated from 4 of the 5 HD. CD19+ and CD19- BM PC were isolated and sequenced for all donors. cDNA (150 pg) was used to generate Illumina-compatible sequencing libraries with the NexteraXT library preparation kit (Illumina, San Diego, CA) per manufacturer’s protocols. All samples were sequenced as one batch to eliminate batch effects. Amplified libraries were hybridized to the Illumina single-end flow cell and amplified using the cBot (Illumina, San Diego, CA). Single end reads of 100nt were generated by the HiSeq2500 (Illumina, San Diego, CA). Raw reads generated from the Illumina base calls were demultiplexed using bcl2fastq version 2.19.0. Data cleaning performed with Trimmomatic-0.36 with the following parameters: “TRAILING:13 LEADING:13 ILLUMINACLIP:adapters.fasta:2:30:10 SLIDINGWINDOW:4:20 MINLEN:355”. Processed/cleaned reads were mapped to the Homo sapiens reference genome (GRCm38 + gencode 17) using STAR_2.6.0c with the following parameters: “–twopassMode Basic –runMode alignReads –genomeDir $ (23) –readFilesIn $ (24) –outSAMtype BAM SortedByCoordinate –outSAMstrandField intronMotif –outFilterIntronMotifs RemoveNoncanonical” (25, 26). Gene level read quantification was derived using the subread-1.6.1 package (featureCounts) with a GTF annotation file (Gencode-28) and the following parameters: “ -s 0 -t exon -g gene_name” and “-M -s 0 -t exon -g gene_name” (27). Differential expression analysis was performed using DESeq2 version 1.40.2 with a p-value threshold of 0.05 at a false discovery rate of 0.05 within R version 4.3.1 (https://www.R-project.org/) (28). An empirical Bayes approach for large-scale hypothesis testing and false discovery rate (FDR) estimation was utilized via the R package ashr version 2.2-63 (29). Counts were normalized using DESeq2 which utilizes a median of ratios methods where counts are divided by sample-specific size factors determined by the median ratio of gene counts relative to the geometric mean per gene. Heatmaps were generated using the Pretty Heatmaps (pheatmap) package version 1.0.12 with *vst* variance-stabilized expression values. Gene ontology analyses were performed on genes with an absolute log2 fold change >1 and adjusted p-value <0.05 using the EnrichR version 3.2 and clusterProfiler version 4.8.3 packages (30).

### ELISA

IFN-α concentration was measured in thawed blood serum and BM supernatant using the Verikine-HS Human IFN-α all subtype ELISA (PBL Assay Science, product 41115), which has an assay range of 1.95 to 125 pg/ml. Lower limit of detection for each IFN-α subtype per manufacturer was as follows: α1 (αD) 0.76, α2a (αA) 0.42, α4a (αM1) 0.43, α5 (αG) 0.51, α6 (αK) 0.33, α7 (αJ1), 0.59, α8 (αB2) 0.82, α10 (αC) 0.65, α14 (αH) 0.51, α16 (αWA) 0.68, α17 (αI) 0.50, and α21 (αF) 1.6 pg/ml. Results were calculated using a basic endpoint protocol and 4-parameter analysis for the standard curve.

### Anti-nuclear antibody immunofluorescence

The ZEUS IFA ANA HEp-2 system (ZEUS Scientific) was used by the clinical laboratory for qualitative and semi-quantitative detection of ANA in thawed serum and BM supernatant from SLE (n=9) and HD (n=7). This is an FDA cleared system utilizing Hep-2 cells and goat anti-human immunoglobulin labeled with FITC. Samples were initially screened at 1:80 dilution. Positive samples at 1:80 were then serially diluted to determine titer. Slides were photographed at 200x magnification with black set at 1000 and white at 3300 on a Zeiss Axioplan Imager fluorescent microscope.

### Mesenchymal stem cell secretome

BMMC were seeded at a cell density of 5–10 × 10^5^ cells/mL in 5 mL MSC medium. MSC medium consisted of HyClone Dulbecco’s Modified Eagle Medium (DMEM; GE Healthcare) supplemented with 10% heat-inactivated fetal bovine serum (FBS, Sigma) and 1% Antibiotic–Antimycotic (i.e. 100 units/mL penicillin, 100 μg/mL streptomycin) in a 25-cm^2^ cell culture flask. Media containing non-adherent cells was removed over the next 2-7 days and replaced with MSC medium. The plastic-adherent (stromal-like) cells were trypsinized and passaged when they reached 80–90% confluence. Supernatants were harvested daily for an entire week from >80% confluent BM-MSC monolayer cultures from passage 2. Supernatants collected from all days were then pooled and centrifuged at ~500 × g for 10 min at 4°C and then at 2000 × g for 30 min at 4°C to remove cell debris. After filtering (0.22 μm Filter System; Corning), supernatants were aliquoted for immediate use or stored at −80°C for subsequent usage.

### SINE inhibitor assay

Stock solutions of KPT-335 (verdinexor, Karyopharm) were prepared at 100μM, 10μM, and 1μM. PBL and BMMC from HD and SLE donors were washed four times with phosphate-buffered saline (PBS) to remove IgG. PBMC and BMMC were cultured in 1:1 MEM 10% FBS: MSC secretome media [protocol adapted from (7)] at 200μl per well in sterile 96-well U bottom plates. PBMCs (200,000-500,000 cells per well in duplicate)/BMMCs (100,000-300,000 cells/well in duplicate) with 0.01μM, 0.05μM, 0.1μM, 0.5μM, 1μM, and 10μM KPT-335 (verdinexor) for 24 hours-4 days. BMMC had an increased frequency of antibody-secreting cells (ASC) so less total BMMC were used per well. For each time point, plates were washed in PBS, spun down, resuspended in RPMI with 10% FBS, and seeded on antibody-coated 96-well MultiScreen Filter plates (Millipore MSIPN4W50) for ASC quantification by ELISPOT assay. Percentage of ASC was determined by enumerating spots in the treated wells normalized by the mean number of spots in untreated wells for each subject. Data was fit using the log of verdinexor concentrations and a variable slope model was used for normalized data and logarithmic concentration of inhibitor [Y=100/(1 + 10^((LogIC50-X)*HillSlope)))]. As the log of 0 is undefined, -10 was input for untreated samples. The Extra Sum of Squares F Test was used to determine if the LogIC50 was different between data sets.

### IgG ELISPOT

ELISPOT filter plates were coated 1 hour to overnight with Human IgG-Fc solution (5ug/ml in 1X PBS, Jackson Immunoresearch, 109-006-098). Excess coating antibody solution was then removed and the plate was blocked with 200μl/well of RPMI with 10% FBS media. The block was then replaced with 200μl of cell suspension and the plate was incubated at 37°C overnight. The following day, plates were washed 3-6x with 1x PBS + 0.1% Tween (PBST). Human IgG-Fc Antibody AP conjugate detection antibody (1:1000 in PBST + 2% bovine serum albumin, Jackson Immunoresearch, 109-055-008) was added at 50ul and incubated 2 hours. Plates were washed 3 time with PBST then developed in 0.1M Tris-HCL (Invitrogen 15568-025) using the Vector Blue AP substrate Kit III (Vector Laboratories, SK-5300). ASC were enumerated on a CTL ImmunoSpot S6 Micro M2 Analyzer (Cellular Technology Ltd, Cleveland, OH).

### Phosphorylation by flow cytometry

MC were freshly isolated from age-, race-, and sex- matched HD and SLE PBL and BM samples. The cells were then treated with either RPMI 1640 media containing 10% FBS and 1% penicillin/streptomycin (Gibco, 15140-22) or media supplemented with 500U/ml of IFN-α2 for 25 minutes at 37°C. Experiments establishing kinetics and concentration of maximal STAT1 phosphorylation for IFN-α2 were previously published (31). After treatment, cells were washed then fixed with 1% formalin for 10 minutes at room temperature. Fixed cells were incubated with Fc block solution (Human TruStain FCx, BioLegend, #422302), surfaced stained with antibodies for 30 minutes at 4°C followed by LIVE/DEAD™ Fixable Aqua Dead Cell Stain Kit (Invitrogen, L34966). Surface stain antibodies include CD3-ALX700 (clone SP34-2, BD 557917), CD11c- BUV395 (clone B-ly6, BD563787), CD14-ALX700 (clone M5E2, BD 557923), CD19-BV605 (clone SJ25C1, Biolegend 363024), CD20-APC-Cy7 (clone 2H7, Biolegend 302314), CD27-BUV737 (clone L128, Biolegend 612829), CD38-BB700 (clone HIT2, BD 566445), IgD-BV421 (clone IA6-2, Biolegend 348226), IgG-BV786 (clone G18-145, BD 564230), IgM-PE-Cy5 (clone G20-127, BD551079) and IgA-APC (clone IS11-8E10, Miltenyi Biotech 130-113-472). Cells were then fixed using BD CYTOFIX for 10 minutes at room temperature followed by 90% BD Phosflow perm buffer III for 20 minutes at 4°C. Two washes were performed before intracellular antibody staining of T-bet-PE-Cy7 (clone 4B10, BioLegend 644824), STAT1-PE (clone 1/STAT1, BD 558537), pSTAT1-Alexa 488 (clone 4A, BD 612596) and immunoglobulins (IgG, IgM and IgA as above) for 45 minutes at 4°C. Following intracellular staining cells were washed and fixed in 1% formalin for 15 minutes at room temperature. An additional 2:1 of 1% BSA/PBS was added and samples were stored at 4°C overnight until flow cytometry. Fold change was calculated by dividing the median fluorescent intensity (MFI) of pSTAT1 for the treated sample by that of the unstimulated sample.

### Anifrolumab labeling

Anifroluamb-fnia is a clinically available human monoclonal antibody against IFNAR1 that was engineered with three heavy chain site mutations to minimize binding IgG Fc receptors. Aliquots of anifrolumab-fnia (Saphnelo, Astra-Zeneca) diluted in 0.9% Sodium Chloride were frozen at -80°C until use. Aliquots were then diluted to 1 mg/ml in molecular grade water and conjugated to Alexa Fluor 647 using the Lightning-Link Rapid Antibody Labeling kit (Novus Biologicals, catalog 336- 0005). A stain index of anifrolumab-Alexa Fluor 647 was calculated to obtain the optimal concentration of 1ug/test to use in flow cytometry. A fluorescence minus one (FMO) control was utilized for gating. Immunostaining was performed using eBioscience FoxP3/Transcription Factor Staining Buffer Set (Invitrogen, catalog 00-5523-00) following the manufacturer’s recommendations. Surface stain antibodies include CD3-ALX700 (clone SP34-2, BD 557917), CD14-ALX700 (clone M5E2, BD 557923), CD19-Super Bright 780 (clone SJ25C1, Invitrogen 78-0198-42), CD27-BUV737 (clone L128, BD 612829), CD38-BV605 (clone HIT2, BD 740401), CD138-PE (clone MI15, BD 552026), CD24-BB515 (clone ML5, BD 564521), Anifrolumab-ALX647, IgD-BV421 (clone IA6-2, Biolegend 348226), and IgG-BUV395 (clone G18-145, BD 564229). Both extracellular and intracellular staining was performed for anti-IgG.

### Statistical analysis

Data were plotted with GraphPad Prism or R Studio with ggplot2 (32) and ggpubr (33) packages. Statistical tests were performed as described in the figure legends.

### Data availability

RNA sequencing data were deposited in the Gene Expression Omnibus database (https://www.ncbi.nlm.nih.gov/geo/) under accession number GSE278120.

## Results

### Experimental strategy

We isolated MC from paired PBL and BM from HD and SLE donors for transcriptomic analysis, flow cytometry, ELISA, and ELISPOT assays (**Figure 1A**). All SLE patients recruited had positive ANA and met 1997 American College of Rheumatology classification criteria for SLE (20) and overall had low disease activity (**Supplementary Table 1**).

Our initial phenotyping strategy (**Figure 1B**) was based on that of previously published work by Lee and Sanz (18) which separated IgD- CD3- CD14- cells into PC populations based on CD19 and CD138 expression. Using this gating strategy, cells were divided into population A (CD19+ CD38 hi CD138-), population B (CD19+ CD38 hi CD138+), population C (CD19- CD38 hi CD138-) and population D (CD19- CD38 hi CD138+) (**Figure 1B**) (34). PB (CD19+ CD27+ CD38 hi) are expected to be in population A while LLPC should reside in the population D (CD19- CD38 hi CD138+). Later, for our bulk RNA-sequencing transcriptomic analysis (**Figure 1C**), CD27 and CD24 were also incorporated as part of the sort to separate into putative LLPC (IgD- CD38 hi CD27 hi, CD138+, CD19- from BM), putative SLPC (IgD- CD38 hi CD27 hi, CD138+, CD19+ from BM), PB (IgD- CD38 hi CD27 hi, CD19+ from PBL), and transitional B cells (CD19+ IgD+, CD27-, CD24 hi, CD38 hi).

### Phenotyping in SLE versus HD bone marrow and blood

**Figure 2:**
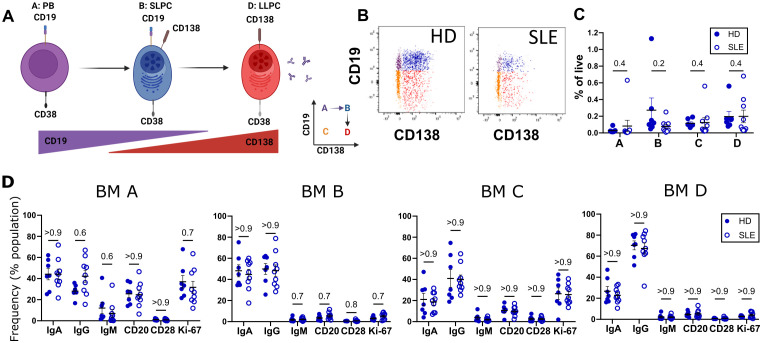
Immunophenotyping of SLE vs. HD BM. BM derived from 9 SLE (○) and 7 HD (⚫) analyzed by flow cytometry. **(A)** Putative differentiation cascade of ASC. A, B and D represent populations from gating in **Figure 1B**. Figure made with BioRender.com **(B)** Flow cytometry gates A (purple), B (blue), C (orange), and D (red) representing BM PC populations for HD and SLE backgated onto CD19 vs CD138 plot. **(C)** Frequency of BM populations A, B, C, and D as a % of live single cells. **(D)** Immunophenotyping of immunoglobulin isotypes (IgG, IgA, IgM) and markers (CD28, CD20, and Ki-67) as % of BM fraction A, B, C or D. Error bars indicate mean ± SEM. Statistics calculated as non-parametric Mann-Whitney Test between SLE and HD groups with q-values reported utilizing a two-stage set up Benjamini, Krieger, and Yekutieli correction.

We utilized SLE (n=9) and HD (n=7) PBMC and BMMC for multiparameter flow cytometric immunophenotyping using the gating strategy shown in **Figure 1B**. PB are the early differentiation state of antibody-secreting cells (ASC) in the blood which during maturation gradually lose CD19 expression while gaining CD138 positivity (**Figure 2A**). HD and SLE BM had a similar distribution of CD38+ PCs in fractions A, B, C, and D based on the CD138 versus CD19 expression (**Figure 2B**) as well as when measured as frequency of live cells (**Figure 2C**). PC represent a low frequency of the overall live BMMC for both SLE and HD (**Figure 2C**). Frequencies of these PC populations were not statistically different between SLE and HD (**Figure 2C**). We did not see any statistically significant differences in the frequencies of IgA, IgM, or IgG expressing PCs when comparing SLE to HD for any of the A, B, C, or D populations from BM (**Figure 2D**). IgA and IgG were expressed at a greater frequency than IgM in all BM PC compartments (**Figure 2D**).

Ki-67, a proliferation marker expressed by cycling cells (35), is thought to distinguish PB from more mature PC. Within the bone marrow compartment, PC populations A and C (CD138-) express Ki-67 (**Figure 2D**). The CD138+ populations in the bone marrow on the other hand did not express Ki-67 (**Figure 2D**, BM B and BM D), consistent with mature PC being in a resting, non-dividing state.

CD28 is a co-stimulatory molecule best known for its role in T cell activation. In LLPC, CD28 has been suggested to contribute to LLPC survival via NF-κB pathways and mitochondrial respiration in mouse models (36). Thus, we were interested in determining if there were differences in levels of expression of CD28 in our autoimmune PC compared to those from HD. We did not see any difference in the frequency of CD28 PC expression (**Figure 2D**) between HD and SLE for any PC compartments.

**Figure 3:**
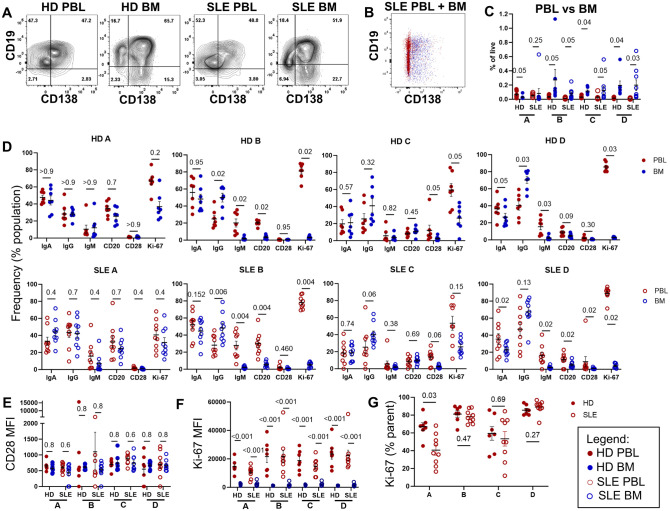
Immunophenotyping of BM and PBL PC. Paired PBL (red) and BM (blue) derived from 9 SLE (○) and 7 HD controls (⚫) analyzed by flow cytometry via the gating strategy shown in **Figure 1B**. **(A)** Representative dot plots of CD19 and CD138 expression of CD14- CD3- IgD- CD24- CD27+ CD38+ PC from PBL and BM from HD and SLE donors. **(B)** Overlay of SLE PBL (red) and BM (blue) CD19 versus CD138 dotplots from single representative SLE subject. **(C)** Frequency of populations A, B, C, and D shown as a % of live single cells, with q-value shown for donor matched PBL vs BM comparison. **(D)** Immunophenotyping of immunoglobulin (IgA, IgG, IgM), CD20, CD28 or Ki-67 markers shown a % of the parent PC fraction. **(E, F)** MFI of PC fraction shown for **(E)** CD28 and **(F)** Ki-67 expression. **(G)** Frequency of Ki-67+ cells in each population of PC within PBL. For all graphs, each point represents data from single donor. Error bars indicate mean ± SEM. Statistics calculated as non-parametric Wilcoxon matched-pairs signed rank test for PBL versus BM comparisons within donors. For all graphs, q-values are reported utilizing a two-stage set up (Benjamini, Krieger, and Yekutieli) correction after either Mann-Whitney (HD vs. SLE) or Wilcoxon matched-pairs signed rank test (PBL vs. BM, matched by donor).

We next compared PBL to BM PCs in both HD and SLE. Most of the CD14- CD3- IgD- CD24- CD27+ CD38+ were CD19+ in PBL (**Figure 3A**). CD138+ PC cells were more prevalent in BM (blue) compared to PBL (red) (**Figure 3B**). For both PBL and BM, PC fractions made up a small percentage of the total live MC (**Figure 3C**). When comparing the PBL and BM compartment, PBL of HD had significantly more of population A, which contains PB. A similar trend is observed in SLE but did not reach statistically significance (**Figure 3C**, red PBL versus blue BM). Populations B (q=0.05 both), C (q=0.04 HD, q =0.05 SLE), and D (q=0.04 HD, q=0.03) were higher in BM (blue) than PBL (red) for both SLE (open circles) and HD (filled circles, **Figure 3C**).

Within the CD19+ CD138- population A, PBL and BM contained similar ratios of immunoglobulins with IgM being found at the lowest frequency for both SLE and HD (**Figure 3D** left). Within the CD138+ B and D populations, BM showed higher frequencies of IgG+ cells while PBL showed higher frequencies of IgM+ cells for both SLE and HD.

CD20 is targeted by the B cell depleting monoclonal therapeutic antibody rituximab. CD20 was expressed on a lower percentage of CD138+ PC in BM compared to PBL for CD19+ fraction B (q=0.02 HD, q=0.004 SLE) and CD19- fraction D (q=0.09 HD, q=0.02 SLE).

We did not see differences in frequency nor level of expression (MFI) for CD28 (**Figures 3D, E**). Interestingly, a small number of cells within all PC populations from PBL expressed Ki-67 (**Figure 3D**). PBL source (as opposed to BM source) of PC predicted higher frequency and expression level of Ki-67, more so than CD19 or CD138 status. Ki-67 expression level was significantly higher by MFI in PBL compared to BM for all PC compartments in both SLE and HD (**Figure 3F**). HD PBL had a higher percentage of Ki-67+ PC in population A compared to SLE PBL (**Figure 3G**).

Thus, the PCs of the PBL were more likely to be dividing and express markers utilized for therapeutic targeting compared to those of the BM. Further, we did not see any major differences in PC phenotype between HD and SLE.

### Lupus plasma cells display an interferon gene signature

To identify pathways that might contribute to PC survival in BM as well as pathways upregulated in autoimmunity, we performed transcriptomic analysis on sorted PBL PB, BM SLPC and BM LLPC from both HD and SLE (**Figure 1C**, sorting scheme in **Supplementary Figure 1A**). We also sorted for transitional B cells, which make up a small percentage of circulating B cells in HD, but are expanded in SLE patients (37). After sorting and RNA quality control, HD BM transitional B cells (n=5), HD LLPC (n=6), HD PBL PB (n=4), HD BM SLPC (n=5), SLE PBL B transitional (n=5), SLE BM transitional B cells (n=5), SLE BM LLPC (n=6), SLE PBL PB (n=6), and SLE SLPC (n=6) transcriptome were sequenced by bulk RNA-seq. We did not have adequate high-quality mRNA from HD PBL transitional B cells to perform RNA-seq.

**Figure 4:**
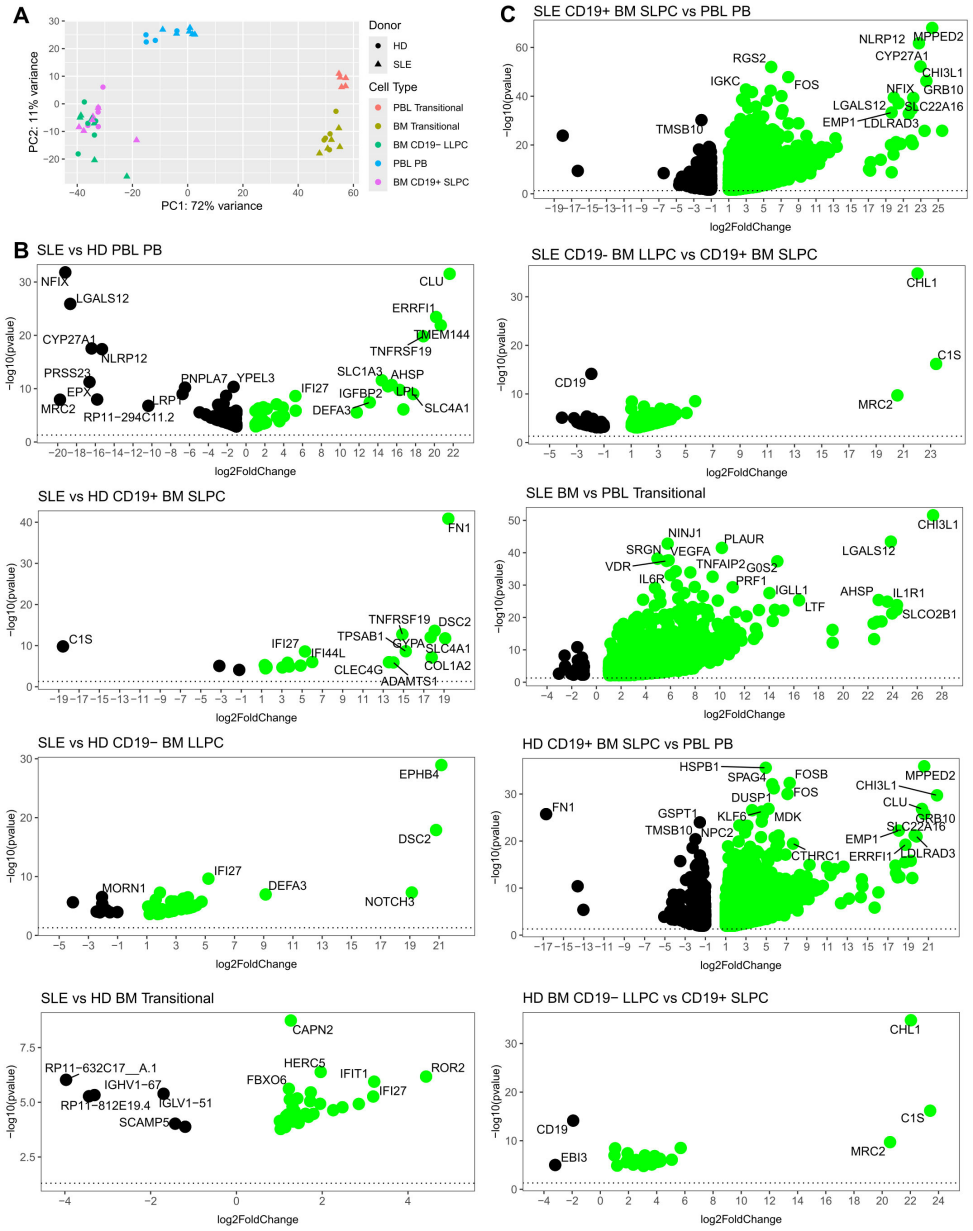
Differential Gene Expression Analysis of SLE and HD BM and PBL PC and transitional B cells. To minimize the impact of sex-associated genes on downstream analysis, a filtering scheme was applied such that genes had to be present in >9 samples and have a count of at least 10 copies. **(A)** Principal Component Analysis (PCA) of normalized expression data. Donor type represented by shape with triangle for SLE and circle for HD. Color denotes cell type. **(B, C)** Volcano plots with top 30 differentially expressed genes annotated for **(B)** SLE vs. HD (left) where green indicates upregulated in SLE and **(C)** BM vs. PBL (right) where green indicates upregulated in BM.

Principal component analysis (PCA) shows a clear separation of PC from transitional B cells (PC1) (**Figure 4A**). Amongst the PC populations, cells from PBL are separated from BM PC (PC2). Cells clustered more distinctly based on cell type or PBL versus BM source than by HD (⚫) or SLE (▲) donor status (**Figure 4A** PCA, sample similarity by Euclidean distance **Supplementary Figure 1B**, and hierarchical clustering analysis in **Supplementary Figure 1C**). SLE versus HD differential expression by cell type showed interferon-associated genes (such as IFI27 and IFI44L) amongst the top 30 upregulated genes (**Figure 4B**).

The comparison between BM SLPC versus PBL PB yielded the highest numbers of differentially expressed genes (DEG, adjusted p-values <0.05) for both HD (1,885 DEG) and SLE (3,061 DEG; **Figure 4C**). SLE BM vs PBL transitional cells (1,428 DEG) showed greater numbers of differentially expressed genes than did SLE vs HD BM transitional cells (35 DEG). BM SLPC versus LLPC had 140 DEG for SLE and 28 DEG for HD. For both SLE versus HD, there were 52 DEG for LLPC, 22 DEG for SLPC, 180 DEG for PBL PB and 35 DEG for BM transitional B cells. Thus, comparisons within cell types of the BM compartment showed less DEG than the BM versus PBL comparisons.

**Figure 5:**
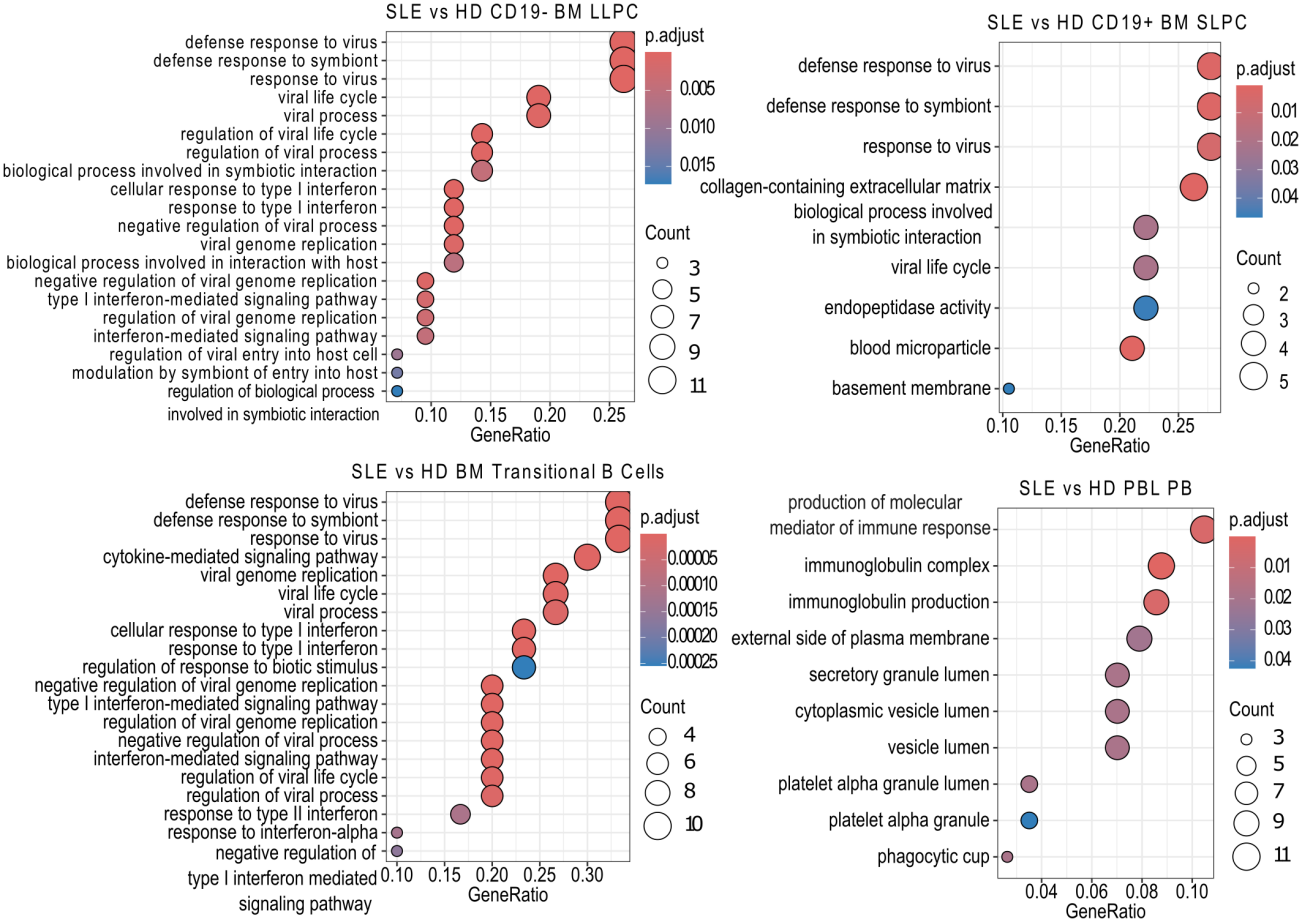
SLE vs HD Over Representation Analysis. Using the *clusterProfiler* enrichGOALL function, pathway enrichment analysis was performed on bulk RNA-sequencing samples differentially expressed genes using the Gene Ontology (GO) database using with an unranked gene list by cell type. The twenty most significantly upregulated pathways are displayed with a p-value cut off of 0.05 and q-value cut off of 0.2 using Benjamini & Hochberg correction (fdr). Size of dot indicates number of genes upregulated in the pathway while color indicates adjusted p-value. GeneRatio indicates the number of genes in the input list associated with the GO pathway divided by the number of input genes.

We then sought to determine which pathways were upregulated in the various subsets using over representation analysis (ORA) and the Gene Ontology (GO) database. Comparing SLE to HD for each PC population revealed that SLE PC enriched for pathways related to viral infection, symbiotic interactions, interferon (BM LLPC and BM SLPC), or production of molecular mediators of the immune response, immunoglobulin complex, and immunoglobulin production (PBL PB) (**Figure 5**). ORA analysis of SLE versus HD BM transitional B cells also showed upregulation of viral and type I and type II interferon pathways in SLE BM transitional B cells (**Figure 5**).

**Figure 6:**
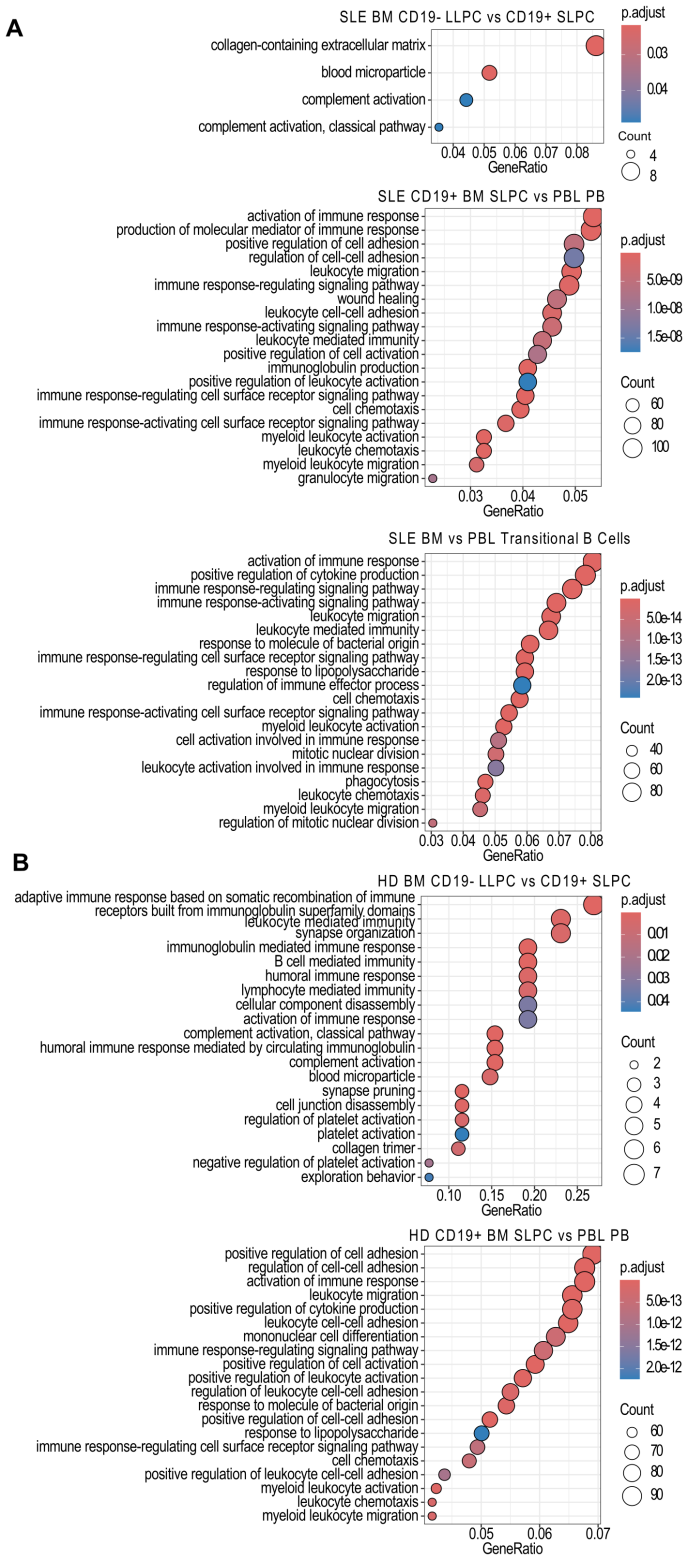
Over Representation Analysis Comparisons Between Cell Subsets. Using the *clusterProfiler* enrichGOALL function, pathway enrichment analysis was performed on bulk RNA-sequencing samples differentially expressed genes using the GO database using with an unranked gene list between **(A)** SLE PC or transitional B cell subsets or **(B)** HD PC subset comparisons. The twenty most significantly upregulated pathways are displayed with a p-value cut off of 0.05 and q-value cut off of 0.2 using Benjamini & Hochberg correction (fdr). Size of dot indicates number of genes upregulated in the pathway while color indicates adjusted p-value. GeneRatio indicates the number of genes in the input list associated with the GO pathway divided by the number of input genes.

Within SLE, only a few pathways (collagen-containing extracellular matrix, blood microparticles, and complement activation) were enriched in BM LLPC versus BM SLPC (**Figure 6A**). In contrast, SLE BM SLPC upregulated pathways related to cell activation and migration compared to PBL PB (**Figure 6A**). Enrichment of these pathways was also seen in HD BM SLPC versus PBL PB (**Figure 6B**). For HD BM PC comparisons, more pathways were significantly enriched in the LLPC including adaptive immune response based on somatic recombination of immune inhibitors built from the immunoglobulin superfamily domains, leukocyte mediated immunity, synapse organization, immunoglobulin mediated immune response, B cell mediated immunity, humoral immune response, and cellular component disassembly (**Figure 6B**). Complement activation and blood microparticle which were seen in SLE was also seen for the LLPC vs. SLPC comparison in HD.

**Figure 7:**
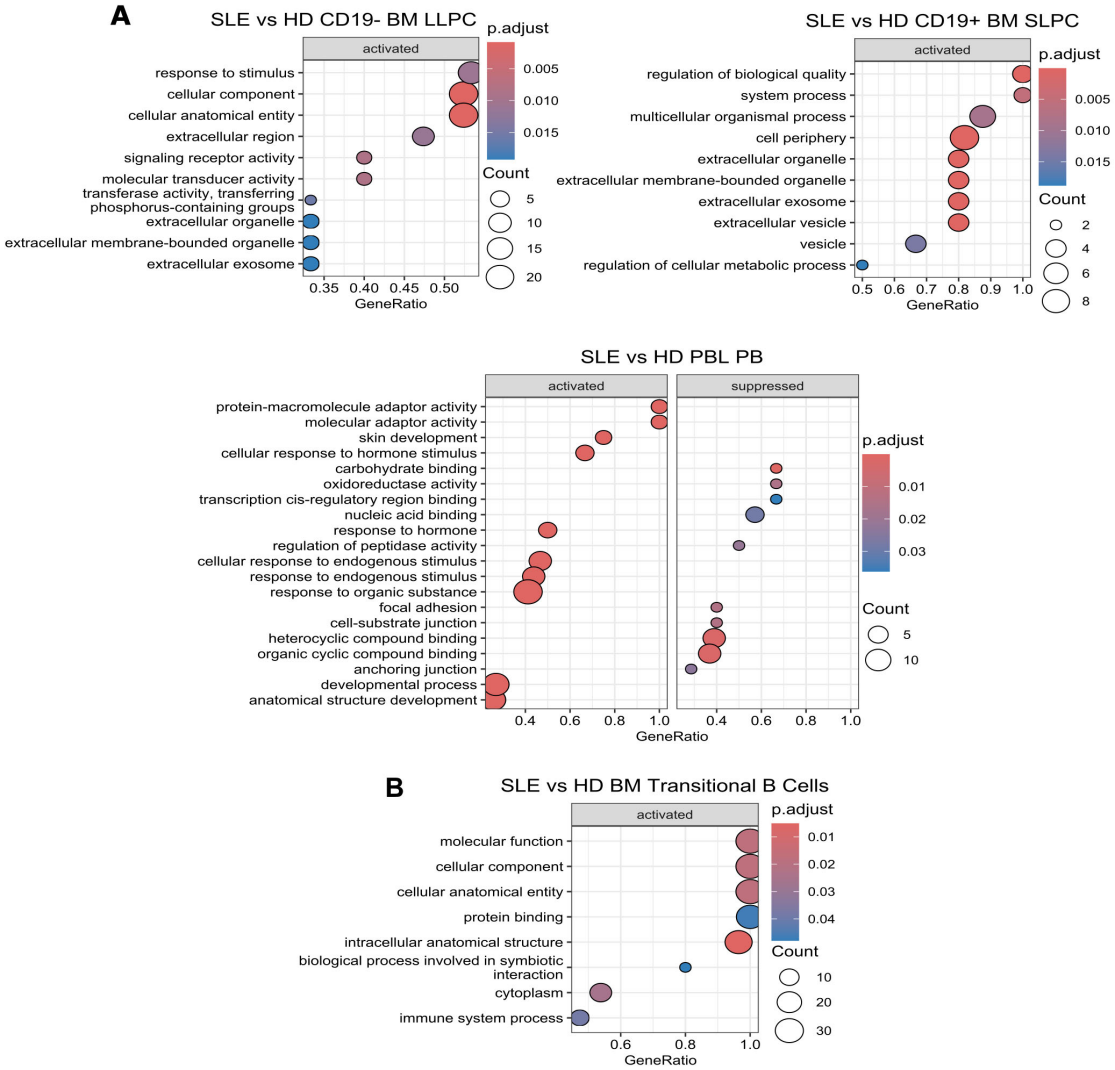
SLE vs HD Gene Set Enrichment Analysis. Using *clusterProfiler*, GSEA analysis was performed on differentially expressed genes with function *gseGO* using the GO database which requires an order ranked gene list on SLE vs. HD comparisons by cell type for **(A)** BM (top) and PBL (bottom) PC subsets and **(B)** BM transitional B cells. Genes were ranked by log2fold change. The twenty most significantly enriched pathways are displayed with a p-value cut off of 0.05 and q-value cut off of 0.2 using Benjamini & Hochberg correction (fdr). Size of dot indicates number of genes upregulated in the pathway while color indicates adjusted p-value. GeneRatio indicates the number of genes in the input list associated with the GO pathway divided by the number of input genes.

**Figure 8:**
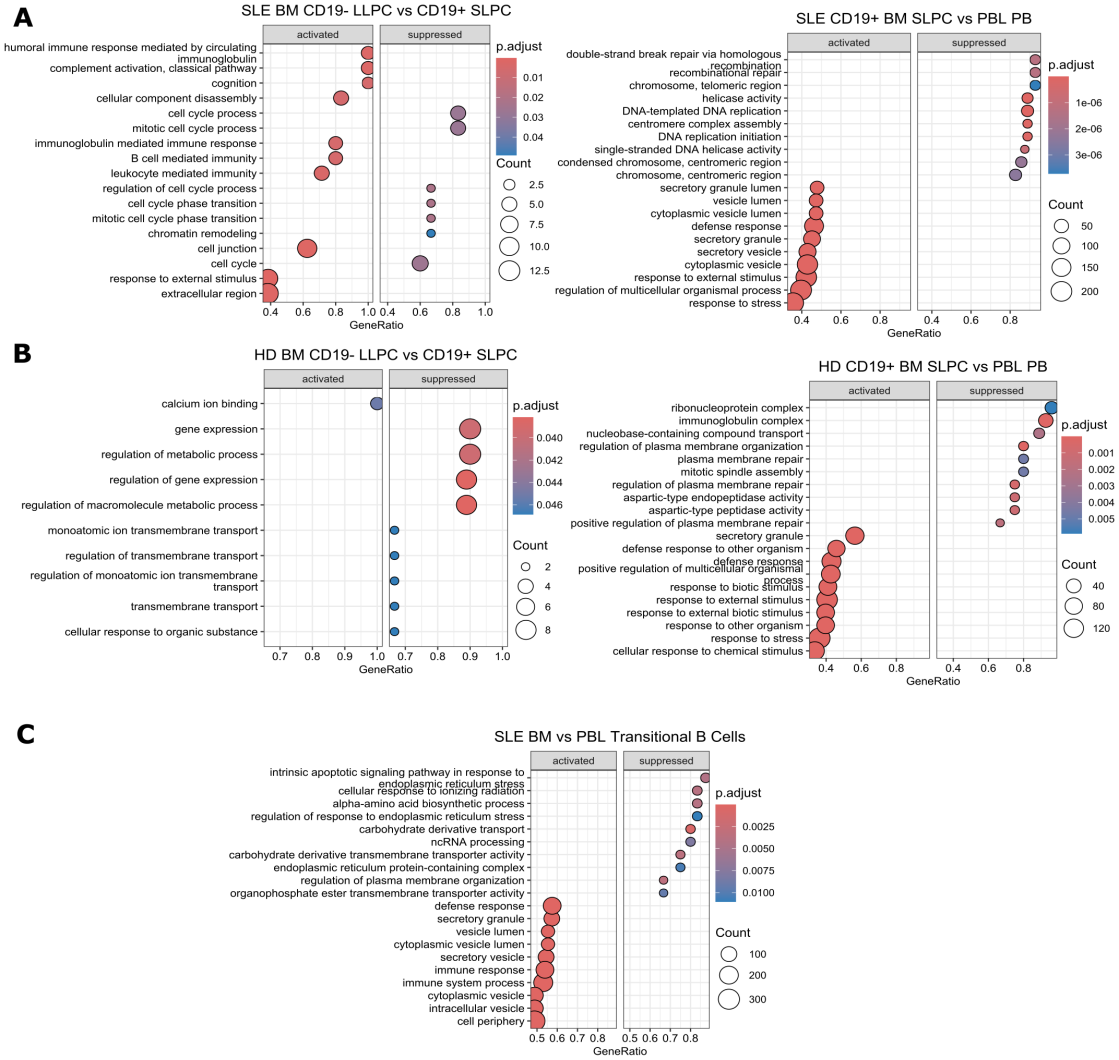
Gene Set Enrichment Analysis Comparisons Between Cell Subsets. Using *clusterProfiler*, GSEA analysis was performed on differentially expressed genes with function *gseGO* using the GO database which requires an order ranked gene list between cell types for comparisons within **(A)** SLE PC subsets, **(B)** HD PC subsets, and **(C)** SLE BM vs. PBL transitional B cells. Genes were ranked by log2fold change. The twenty most significantly enriched pathways are displayed with a p-value cut off of 0.05 and q-value cut off of 0.2 using Benjamini & Hochberg correction (fdr). Size of dot indicates number of genes upregulated in the pathway while color indicates adjusted p-value. GeneRatio indicates the number of genes in the input list associated with the GO pathway divided by the number of input genes.

We repeated the analysis using Gene Set Enrichment Analysis (GSEA)– a method different from ORA in that it requires a ranking of differentially expressed genes by log 2-fold change (**Figures 7**, **8**). Pathways upregulated in SLE LLPC or SLPC when compared to HD LLPC or SLPC by GSEA showed broad biological pathways such as response to stimulus and regulation of biological quality (**Figure 7A**). For BM transitional B cells, GSEA also identified broad biological pathways as upregulated in SLE (**Figure 7B**). More pathways were enriched when comparing SLE to HD PB including both activated (e.g. adaptor activity, response to endogenous stimulus) and suppressed pathways (e.g. carbohydrate binding, oxidoreductase binding, nucleic acid binding) (**Figure 7A**).

Within SLE, BM LLPC activate humoral immune response and complement activation-related pathways while suppressing cell cycle-related processes compared to SLPC (**Figure 8A** left). SLE BM SLPC upregulate secretory processes while downregulating DNA repair pathways (**Figure 8A** right). For HD, LLPC activated pathways included calcium ion binding whereas suppressed pathways included regulation of gene expression pathways. (**Figure 8B** left). BM SLPC from HD upregulate pathways involved in response to external stimuli and downregulate plasma membrane repair processes, ribonucleoprotein complex, and immunoglobulin complex as compared to PBL PB (**Figure 8B** right). When compared to PBL transitional B cells, SLE BM transitional B cells activate pathways for defense response while suppressing stress response pathways such as intrinsic apoptotic signaling pathway in response to endoplasmic reticulum stress and cellular response to ionizing radiation (**Figure 8C**).

Overall, the transcriptomic differences between PCs were most pronounced between BM and PBL rather than between SLE and HD comparisons. SLE BM LLPC had some enrichment in activation and cellular process pathway compared to BM SLPC. Comparisons between SLE and HD cells showed upregulated viral or IFN pathways in the SLE BM.

### Interferon-α detectable in bone marrow

**Figure 9:**
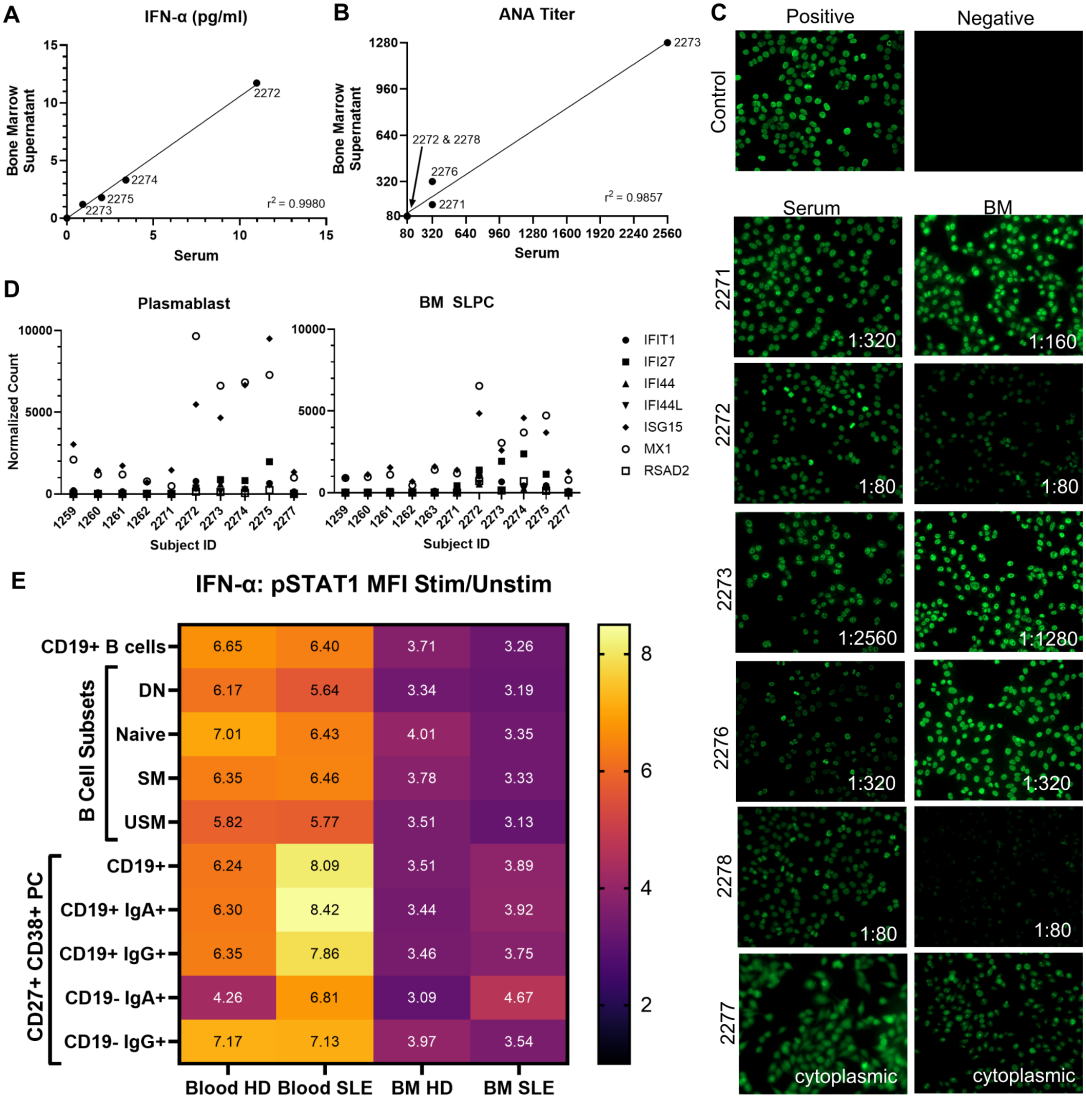
PBL versus BM Relationship with IFN-α and ANA. **(A)** Serum and BM IFN-α levels correlate (spearman r =1, p=<0.0001, n=16, 7 HD, 9 SLE). **(B)** Serum and BM ANA titer correlate (spearman 0.9733, p=0.033). For A-B, subject IDs of positive samples and simple linear regression coefficient shown. **(C)** ANA immunofluorescence in serum and BM supernatant (7 HD, 9 SLE). Lowest titer by which fluorescence still visible shown for positive samples. **(D)** IFN Stimulated Genes normalized counts from bulk RNA-seq from CD19+ BM SLPC and PBL PB. HD represented by 1259 to 1263 and SLE 2271 to 2277. **(E)** SLE and HD PBL and BM B cells and PC signal in response to IFN-α stimulation. Heat map of mean pSTAT1 MFI IFN-α Stimulated/Unstimulated Fold Change (n=6 HD, n=6 SLE) by flow cytometry shown. All CD19+ subsets had q-value of 0.03 between PBL and BM pSTAT1 fold change using Wilcoxon matched-pairs signed rank test with a two-stage step up false discovery approach by Benjamin, Krieger and Yekutiel. Differences between CD19- IgA+ or CD19- IgG+ were not significant for SLE versus HD or paired PBL versus BM tests. Mann-Whitney tests used between HD and SLE for PBL or BM subsets were not significant.

A type I IFN signature is found in circulating PBL in SLE (38). IFN-α is a type I interferon cytokine which causes differentiation of B cells to PCs (39). Since response to type I IFN gene signature was upregulated in SLE LLPC when compared to HD LLPC, we next asked if type I IFN could be detected in matched PBL serum and BM supernatant. As IFN-α is the prototypical IFN most frequently implicated in SLE, we measured IFN-α (n=7 HD, n=9 SLE) with an all subtype ELISA which could detect 12 different isoforms of IFN-α. We detected IFN-α in 4 out of the 9 SLE samples analyzed (IFN-α range of 0-10.98pg/ml, mean ± SEM of 1.93 ± 1.20pg/ml for serum, range of 0-11.71 pg/ml, mean of 2.00 ± 1.27pg/ml for BM supernatant). IFN was not detectable in the HD samples. The BM IFN-α levels highly correlate with blood IFN-α levels (spearman correlation of 1 with p<0.0001) with a simple linear regression coefficient (r^2^) of 0.998 (**Figure 9A**). Samples with detectable IFN-α had higher levels of IFN response genes such as MX1 as measured by bulk RNA-seq normalized counts (shown for BM SLPC and PB, **Figure 9D**). There was a statistically significant difference (Mann-Whitney, data not shown) in normalized count between HD and SLE for BM SLPC for IFIT1 (p = 0.009), IFI27 (p=0.009), and IFI44 (p=0.03).

### Antinuclear antibodies detectable in bone marrow supernatant

All SLE patients had a history of prior positive clinical ANA to be enrolled in the study. Blood serum and BM supernatant from the time of study enrollment were sent to the clinical lab for ANA titer measurement. We detected ANA in 5/9 of the SLE patients and none of the 7 HD sera. The same subjects with positive ANA in the serum, also had positive ANA in the BM supernatant as shown in **Figures 9B, C**. Of note, one SLE patient had positive cytoplasmic staining but negative ANA staining (subject 2277). We did not find a correlation of positive ANA with IFN-α level (data not shown).

### SLE blood and bone marrow plasma cells phosphorylate STAT1 in response to IFN-α

To determine if SLE PCs remain responsive to IFN, we stimulated PBMC and BMMC with IFN-α then measured pSTAT1 (part of the type I IFN signaling cascade) via flow cytometry. BM PC from both SLE (n=6) and HD (n=6) were responsive to IFN-α as measured by the ratio of stimulated to unstimulated pSTAT1 MFI (**Figure 9E**). Consistent with our previous phenotyping, fewer CD19- IgA+ or CD19- IgG+ LLPC were found in PBL compared BM. Thus for PBL, we only had adequate CD19- PC cell numbers to calculate pSTAT1 fold change for n=4 for IgA+ in HD, n=5 for IgG+ in HD, n=5 for IgA+ in SLE, and n=5 for IgG+ in SLE whereas pSTAT1 fold change was calculated for all n=6 SLE and n=6 HD in the BM samples. PBL PC and PBL B cells were more responsive to IFN-α than BM PC and BM B cells from both SLE and HD donors (q = 0.03). SLE PBL CD19+ PB had a higher mean fold change in pSTAT1 in response to IFN-α than HD PBL, but this was not statistically significant. Thus, PCs from the SLE BM display an IFN gene signature (as seen in our transcriptomics analysis), IFN-α is detectable in some SLE BM supernatant, and PC remain functionally responsive to IFN-α.

### Anifrolumab binds B cells and plasma cells

**Figure 10:**
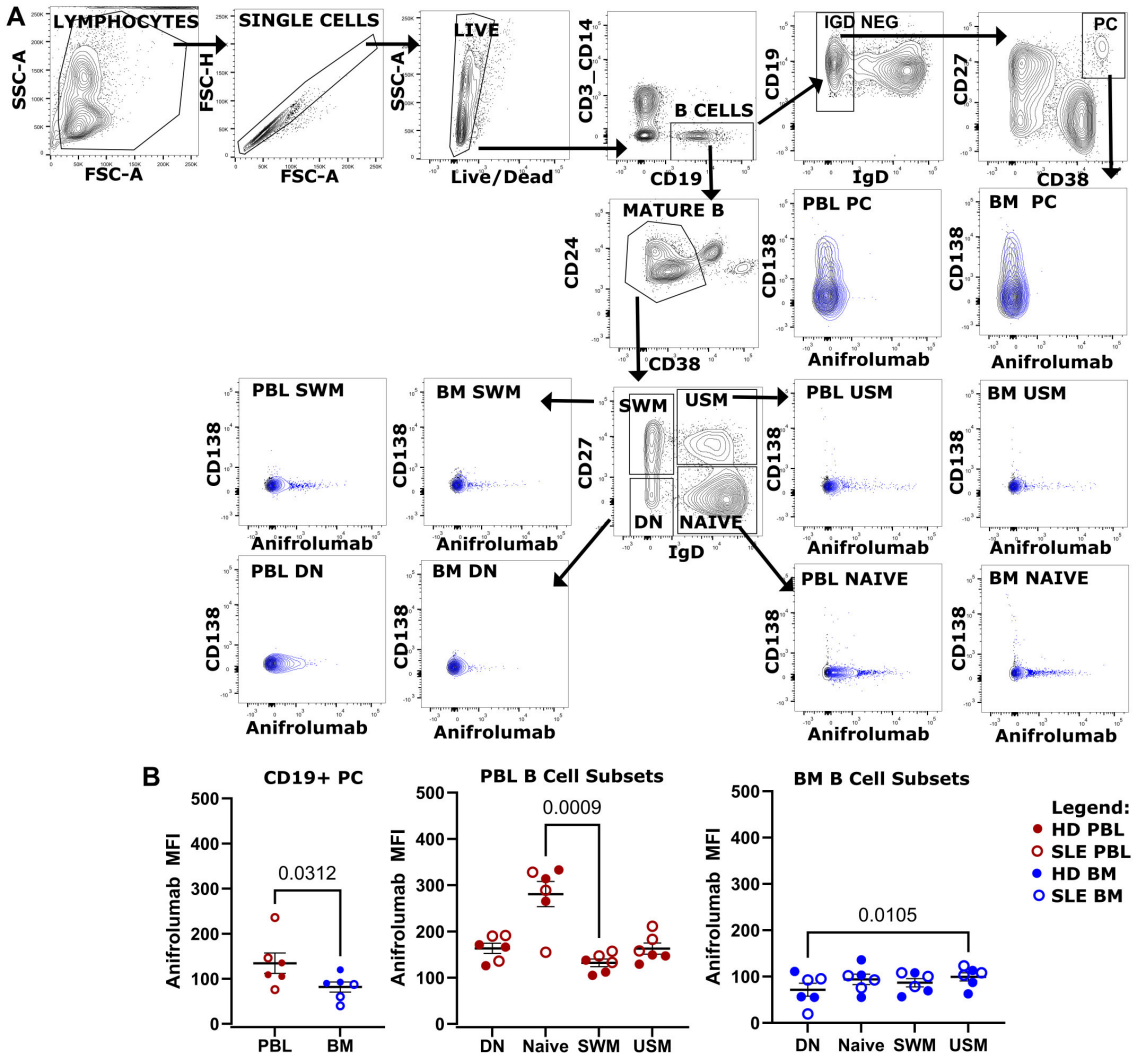
Anifrolumab binds B cell and PC. **(A)** Flow cytometry phenotyping of canonical B cell subsets and CD19+ PC using a panel containing fluorescently labeled anifrolumab using gating strategy shown. Overlay shown of anifrolumab containing panel (blue) overlaying fluorescence minus 1 control (black) which did not include anifrolumab. BM and PBL from same donor shown. **(B)** Anifrolumab MFI of CD19+ PC in PBL vs BM (left), PBL B cell (middle, red) and BM B cell subsets (right, blue) for pooled HD (○, n=3) and SLE (⚫, n=3). Wilcoxon test p-value shown for PC PBL vs BM. Friedman test with Dunn’s multiple comparison test q-values shown for canonical B cell subsets.

Type I IFN is a therapeutic target in SLE, which is blocked with the type I receptor-blocking FDA-approved monoclonal antibody anifrolumab that binds to IFNAR1. We asked if this drug could bind to mature B cell subsets and PC in the PBL and BM (**Figure 10**). Fluorescently-labeled anifrolumab was included in a flow cytometry panel to immunophenotype B cells and PCs from BM and PBL (**Figure 10A**) from HD (n=3, ⚫) and SLE (n=3, ○) donors. Anifrolumab MFI was higher in CD19+ PCs circulating in the PBL compared to those of the BM (**Figure 10B** left). Compared to PC, higher anifrolumab MFI was measured on B cells subsets. Amongst canonical B cell subsets, circulating naïve B cells from the PBL showed the highest anifrolumab binding (**Figure 10B**). Thus, PBL naïve B cells may be preferentially targeted by anifrolumab compared to the B cells or PC of the BM.

### Bone marrow-derived plasma cells have enhanced survival compared to blood plasmablasts

**Figure 11:**
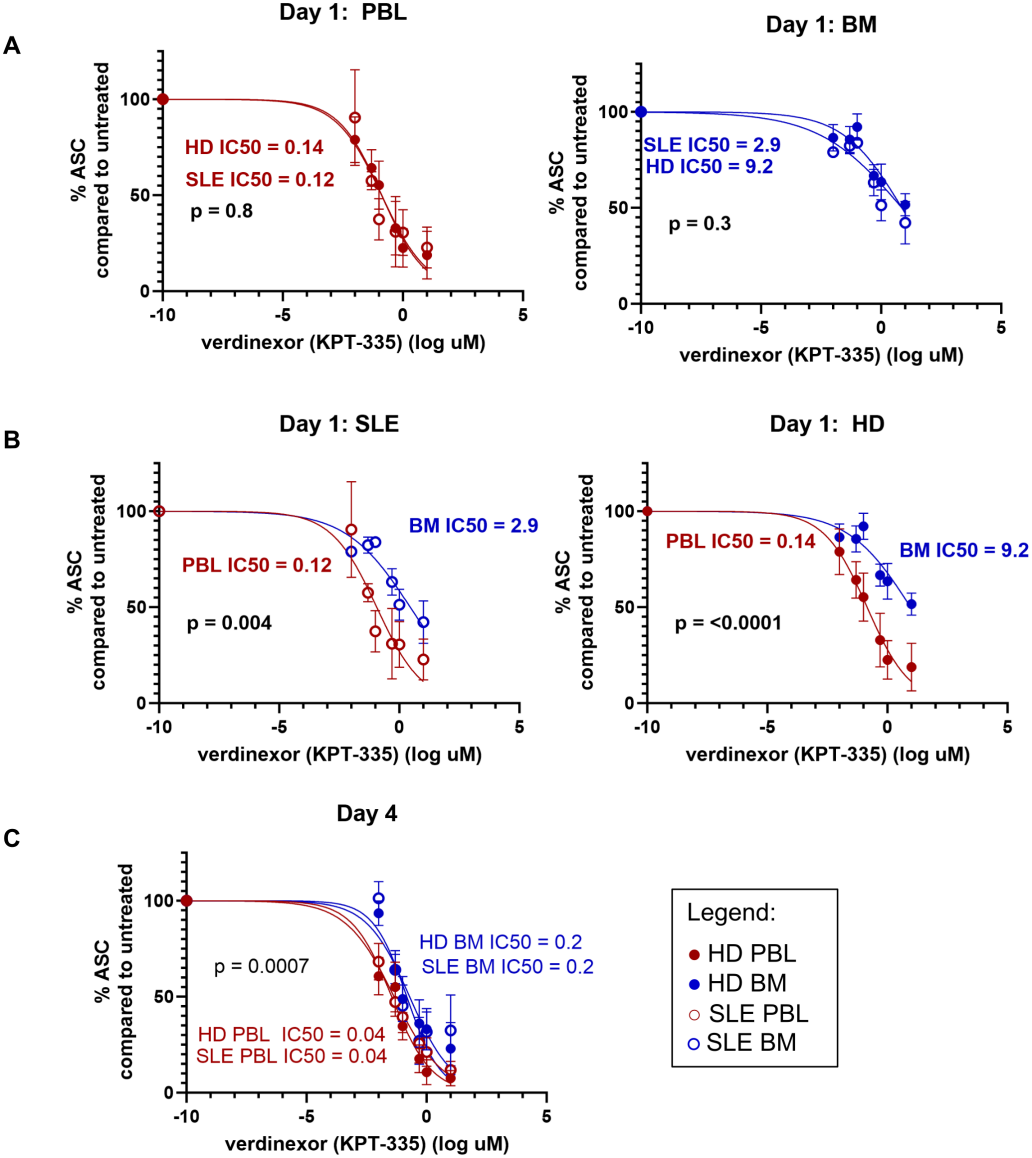
BM ASC have enhanced survival compared to PBL ASC. Surviving IgG+ ASC were enumerated by IgG ELISPOT after *in vitro* treatment of MC with SINE inhibitor KPT-335. Dose response curves for **(A)** Day 1 HD (⚫) vs. SLE (○) for PBL (red, left) and BM (blue, right); **(B)** Day 1 BM vs. PBL for SLE (left, ○) and HD (right, ⚫) and **(C)** Day 4 all sources. The Extra Sum of Squares F Test was used to determine if the LogIC50 was different between data sets. X data was normalized to the mean number of ASC detected in the untreated wells for each subject. Data was fit using the log of verdinexor concentrations with a variable slope model for normalized data and logarithmic concentration of inhibitor [Y=100/(1 + 10^((LogIC50-X)*HillSlope)))]. As log of 0 is undefined, -10 was input for untreated samples.

Cytokines contribute to PC survival in the BM. In SLE, PC exert pathogenic effects through the production of autoantibodies. Lupus nephritis is ameliorated in pre-clinical SLE murine models via a reduction in IgG and anti-dsDNA ASC when mice are treated with SINE inhibitors such as verdinexor (40). In humans, SINE inhibition (selinexor) is FDA-approved for the treatment of refractory multiple myeloma, a malignant PC dyscrasia. Therefore, we hypothesized that SINE inhibition would also exert an inhibitory effect on human PCs derived from SLE patients and thus represent a potential therapeutic for autoimmune disease. MC were isolated from the PBL and BM of both HD and SLE patients for *in vitro* treatment with SINE inhibitor KPT-335 (verdinexor). IgG-secreting PC were enumerated by ELISPOT after 1 and 4 days of verdinexor treatment at a range of concentrations (0 to 10μM). Half-maximal inhibitory concentration (IC50) was calculated and compared by the Extra Sum of Squares F Test. Increasing concentrations of SINE inhibitor led to more cell death for all sources of ASC. There was no difference in ASC survival between SLE and HD as measured by IC50 for either BM or PBL-derived MC (**Figure 11A**, p=0.4 for PBMC, p=0.3 for BMMC at day 1). However, we observed a statistically significant difference in IC50 when PBMC were compared to BMMC (**Figure 11B**, p=0.004 for SLE, p=<0.001for HD at day 1) suggesting that BM PCs were more resistant to SINE inhibition than PBL PB. The IC50 was also significantly different on day 4, but at a lower drug concentration (**Figure 11C**). Thus, verdinexor is effective at suppressing antibody secretion *in vitro* and might represent a promising SLE therapeutic target. However, PBL PB are more sensitive to verdinexor than BM PC, who show some protection from cell death, perhaps due to factors that sustained them in the BM niche.

## Discussion

Here we characterize and compare the PC compartments in PBL versus BM in SLE patients and HD using BM aspirate samples from human subjects, an understudied compartment. We find that PC from SLE and HD have similar distribution of CD38 and CD138 expression in the blood. It is important to note that our SLE cohort is of relatively low disease activity which might explain why we do not see increased frequencies of CD138+ cells in the periphery as has been recently reported by other groups utilizing SLE patients with higher SLEDAI (41). As expected, both SLE and HD have increased frequency of CD138+ cells in BM, a compartment which contains long-lived, mature, terminally differentiated PC. Although CD138+ cell frequency is greatly reduced in the blood, they are still present. One possibility for the presence of CD138+ PC in the PBL is that these PC egress from BM and recirculate. However, timestamp experiments in mice suggest that LLPC of the BM are not displaced into the periphery by newly generated PC nor is the frequency of newly generated PC influenced by available space in the BM niche (42, 43). The presence of CD138+ PC in the blood is explained by a model in which BM LLPC are CD19+ PB that lose CD19 expression as they differentiate and migrate toward the BM (41). Of the cells that differentiate into PC, it is likely only a fraction become LLPC (42).

Ki-67 is a proliferation marker expressed by PB in both HD and SLE (35). Interestingly, while there are few CD138+ PC in blood, those present express Ki-67, suggesting that even these more mature cells may also be dividing in the blood. Ki-67 was not seen on the PC in the BM, consistent with these BM cells being in a resting non-dividing state. Overall, this is consistent with CD19- PC being expanded along a continuum in the periphery and not within the BM as reported by others (41).

As has been shown for many other SLE cell types (44, 45) including circulating PC (46) and tonsil-derived PC (39), our transcriptomic analysis identified pathways related to IFN response among the top 20 pathways upregulated in SLE BM compared to HD when using an unranked gene list (ORA analysis). This was true for B transitional cells [as we have previously shown (47)] as well as for SLPC and LLPC derived from SLE BM. The presence of a type I IFN gene signature was in agreement with a recent paper that also found an upregulated type I IFN signature in SLE BM PCs in a Chinese cohort (48), who felt it was associated with dysregulated B cell lymphopoiesis which had also been suggested by our prior work (49). We have previously shown that neutrophils from SLE BM are a potential source of IFN-α through production as well as stimulation of plasmacytoid dendritic cells (49). As would be expected based on the ORA analysis that identified type I IFN pathway upregulation, we detected IFN-α protein by ELISA in both serum and BM supernatant- results of which were highly correlated. However, we were only able to find IFN-α protein in a subset of our cohort which could be due to the low disease activity or due to the heterogenous presentation of SLE. While IFN-α is commonly associated with SLE, it is not the only IFN that can produce a type I IFN gene signature. In fact, IFN-β has long been known to be produced by bone marrow stromal cells (50, 51) and would not have been measured by our all subtype IFN-α ELISA.

If instead a log2 fold change ranked gene list was used (GSEA) for gene expression pathway analysis, the IFN pathways were not among the top pathways, which were instead dominated by more general extracellular components and signaling pathways. The differences between the two pathway analyses are that ORA utilizes the list of genes with significant differences by differential expression analysis without taking into account the degree of expression so long as the threshold set for statistical significance is met (a gene set). From our data, it is difficult to know whether higher fold change is more biologically significant than a smaller fold change and this may be different depending on the gene. Interestingly, IFN pathways were not among the differentially expressed pathways between SLE and HD for PBL PBs by ORA analysis. Instead, we saw immunoglobulin production pathways and vesicle and secretory lumen pathways.

A limitation of our study is that from our panel, we cannot tell the specificity of these cells or which are autoreactive. While our SLE patients all made autoantibodies to be diagnosed with SLE, our cohort was one of small numbers and low disease activity given the challenges of recruiting for BM aspiration of SLE patients with high disease burden. Furthermore, all patients were on immune-modulating therapies such as hydroxychloroquine, mycophenolate, and glucocorticoids. Despite these limitations, we did identify autoreactive antibodies in the BM supernatant of a subset of patients. As seen with IFN-α protein levels, when we detected ANA by immunofluorescence in the BM, we also found ANA in the serum, though it is difficult to determine the source of either from this data.

True to clinical treatment experience in other disorders (52), BM PCs proved to be more difficult targets when we used a SINE inhibitor, regardless of HD or SLE source. One possibility is that PBL PCs are more likely to be in a proliferative state than BM PCs, consistent with our flow cytometry results for Ki-67 and the upregulation of centromere complex and mitotic spindle pathways in PBL-derived PCs, and thus more sensitive to this therapeutic. SINE drugs are known to cause cell cycle arrest and induce anti-proliferative genes in susceptible cells (53). It remains possible these differences in SINE sensitivity between BM and PB PC may be able to be overcome with increased concentrations of selinexor. Indeed, SINE treatment in a mouse model of SLE was efficacious clinically and reduced BM PCs, though these effects were more related to disruption of PC generation and migration than survival (40).

Our study raises the possibility that anifrolumab has the potential to act on BM PCs by targeting the type I IFN receptor, though to a lesser degree than circulating B cells. In mice, elevated IFN-α serum levels have been shown to overcome immune tolerance checkpoints in the B cell lineage leading to the production of autoantibodies (46, 54). Thus, blocking IFN-α could reduce autoantibody levels. Our data corroborates that anifrolumab does indeed bind B cells and to a lesser degree PCs including those in the BM and these cells are responsive to IFN as shown by phosphoflow. The presence of an IFN gene signature in SLE BM PCs could be due to IFN exposure both during PC generation and in the BM microenvironment. The functional outcome of IFN activation and anifrolumab treatment on BM PCs remains to be elucidated.

Our data demonstrate the challenges in targeting BM PCs, particularly LLPC in SLE. Further, our study highlights a role for IFN in shaping the SLE BM PC compartment.

## Data Availability

RNA sequencing data can be accessed in the Gene Expression Omnibus database under accession number GSE278120. The GEO online repository can be found at: https://www.ncbi.nlm.nih.gov/geo/query/acc.cgi?acc=GSE278120.

## Supplementary material

Supplementary Figure 1 Transcriptomic profiling of PC and transitional B cells from SLE and HD BM and PBL. **(A)** Flow cytometry sort strategy for isolating transitional B cells and PC for bulk RNA sequencing transcriptomic analysis. **(B)** Overall sample similarity represented as heatmap of Euclidean distance between samples using a variance stabilizing transformation on bulk RNA-sequencing count data. **(C)** Hierarchical clustering heat map. Top dendrogram shows sample clustering with transitional cells clustering on the left, BM CD19+ SLPC and BM CD19- LLPC in the middle, and PBL PB on the right. The top 20 mRNA expressed are annotated.

Supplementary Table 1 Donor Demographics. Demographics of HD and SLE donors sorted for bulk RNA-sequencing and used for flow cytometry phenotyping. For SLE patients, medications at time of study, serology titer (ever positive), 1997 ACR SLE classification criteria (ever positive), and Systemic Lupus Disease Activity Index (SLEDAI-2K within 30 days) shown. H = Hispanic, NH = non-Hispanic, AA = African American, and C = Caucasian.

Supplementary Table 2 Metadata. Metadata for the 47 samples used for bulk RNA-sequencing.
