# Supplementary figures and images for: Interferon activation in bone marrow long-lived plasma cells in systemic lupus erythematosus
Source: Front Immunol. 2025 Jan 10;15:1499551. doi: 10.3389/fimmu.2024.1499551 (PMC11757124; doi:10.3389/fimmu.2024.1499551)

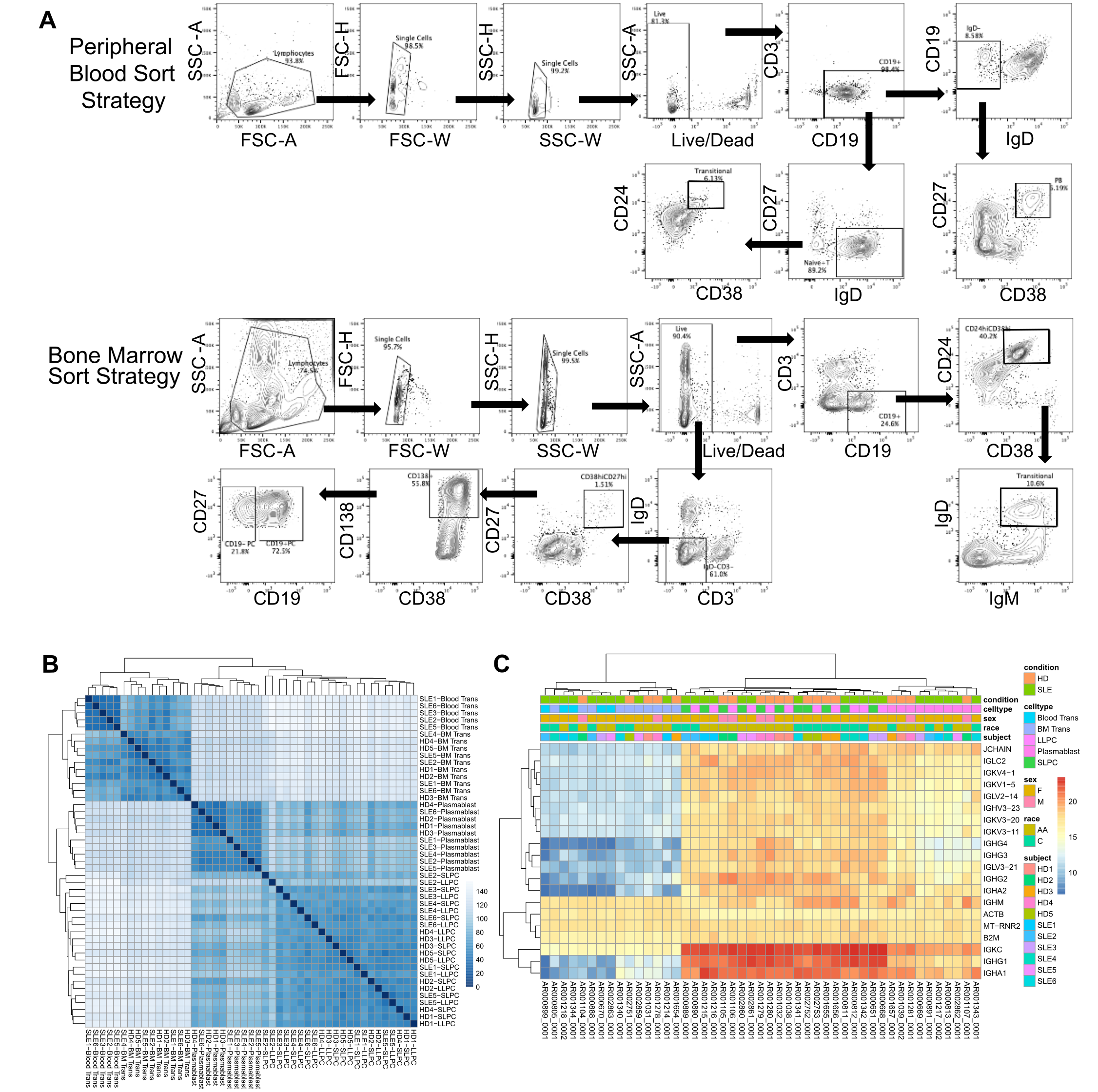

Supplement: Supplementary Figure 1 — Transcriptomic profiling of PC and transitional B cells from SLE and HD BM and PBL. (A) Flow cytometry sort strategy for isolating transitional B cells and PC for bulk RNA sequencing transcriptomic analysis. (B) Overall sample similarity represented as heatmap of Euclidean distance between samples using a variance stabilizing transformation on bulk RNA-sequencing count data. (C) Hierarchical clustering heat map. Top dendrogram shows sample clustering with transitional cells clustering on the left, BM CD19+ SLPC and BM CD19- LLPC in the middle, and PBL PB on the right. The top 20 mRNA expressed are annotated. [file SupplementaryFile1.tiff]
